# Possible association of 16p11.2 copy number variation with altered lymphocyte and neutrophil counts

**DOI:** 10.1038/s41525-022-00308-x

**Published:** 2022-06-17

**Authors:** Giuliana Giannuzzi, Nicolas Chatron, Katrin Mannik, Chiara Auwerx, Sylvain Pradervand, Gilles Willemin, Kendra Hoekzema, Xander Nuttle, Jacqueline Chrast, Marie C. Sadler, Eleonora Porcu, 16p11.2 Consortium, Katrin Männik, Damien Sanlaville, Caroline Schluth-Bolard, Cédric Le Caignec, Mathilde Nizon, Sandra Martin, Sébastien Jacquemont, Armand Bottani, Marion Gérard, Sacha Weber, Aurélia Jacquette, Catherine Vincent-Delorme, Aurora Currò, Francesca Mari, Alessandra Renieri, Alfredo Brusco, Giovanni Battista Ferrero, Yann Herault, Bertrand Isidor, Brigitte Gilbert-Dussardier, Evan E. Eichler, Zoltan Kutalik, Alexandre Reymond

**Affiliations:** 1 Center for Integrative Genomics, University of Lausanne, Lausanne, Switzerland; 2 Department of Biosciences, University of Milan, Milan, Italy; 3 Service de génétique, Hospices Civils de Lyon, Lyon, France; 4 University of Lyon, Université Claude Bernard Lyon 1, CNRS UMR-5310, INSERM U-1217, Institut NeuroMyoGène, F-69008 Lyon, France; 5 Department of Computational Biology, University of Lausanne, Lausanne, Switzerland; 6 Center for Primary Care and Public Health, University of Lausanne, Lausanne, Switzerland; 7 Swiss Institute of Bioinformatics, Lausanne, Switzerland; 8 Department of Genome Sciences, University of Washington, Seattle, WA USA; 9 Center for Genomic Medicine, Massachusetts General Hospital, Boston, MA USA; 10 Department of Neurology, Harvard Medical School, Boston, MA USA; 11 Program in Medical and Population Genetics and Stanley Center for Psychiatric Research, Broad Institute, Cambridge, MA USA; 12 University of Strasbourg, CNRS, INSERM, PHENOMIN-ICS, Institute of Genetics and Molecular and Cellular Biology, Illkirch, France; 13 Service de Génétique Médicale, CHU de Nantes, Nantes, France; 14 Service de Génétique, CHU de Poitiers, Poitiers, France; 15 Howard Hughes Medical Institute, University of Washington, Seattle, WA USA; 16 Centre Hospitalier Universitaire Vaudois, University of Lausanne, Lausanne, Switzerland; 17 University of Montreal, Montreal, Canada; 18 Department of Genetic Medicine and Development, University of Geneva Medical School, Geneva, Switzerland; 19 Service de Génétique Medicale, CHU de Caen, Caen, France; 20 Service de Génétique Clinique, CHU Paris-GH La Pitié Salpêtrière, Paris, France; 21 Service de Génétique Clinique, CHU de Lille, Lille, France; 22 Medical Genetics, University of Siena, Siena, Italy; 23 Department of Medical Sciences, University of Turin, Turin, Italy; 24 Department of Clinical and Biological Sciences, University of Turin, Turin, Italy; 25 Present Address: Health 2030 Genome Center, Foundation Campus Biotech, Geneva, Switzerland

**Keywords:** Haematological diseases, Genetic association study, Medical genetics

## Abstract

Recurrent copy-number variations (CNVs) at chromosome 16p11.2 are associated with neurodevelopmental diseases, skeletal system abnormalities, anemia, and genitourinary defects. Among the 40 protein-coding genes encompassed within the rearrangement, some have roles in leukocyte biology and immunodeficiency, like *SPN* and *CORO1A*. We therefore investigated leukocyte differential counts and disease in 16p11.2 CNV carriers. In our clinically-recruited cohort, we identified three deletion carriers from two families (out of 32 families assessed) with neutropenia and lymphopenia. They had no deleterious single-nucleotide or indel variant in known cytopenia genes, suggesting a possible causative role of the deletion. Noticeably, all three individuals had the lowest copy number of the human-specific *BOLA2* duplicon (copy-number range: 3–8). Consistent with the lymphopenia and in contrast with the neutropenia associations, adult deletion carriers from UK biobank (n = 74) showed lower lymphocyte (*P*adj = 0.04) and increased neutrophil (*P*adj = 8.31e-05) counts. Mendelian randomization studies pinpointed to reduced *CORO1A*, *KIF22*, and *BOLA2-SMG1P6* expressions being causative for the lower lymphocyte counts. In conclusion, our data suggest that 16p11.2 deletion, and possibly also the lowest dosage of the *BOLA2* duplicon, are associated with low lymphocyte counts. There is a trend between 16p11.2 deletion with lower copy-number of the *BOLA2* duplicon and higher susceptibility to moderate neutropenia. Higher numbers of cases are warranted to confirm the association with neutropenia and to resolve the involvement of the deletion coupled with deleterious variants in other genes and/or with the structure and copy number of segments in the CNV breakpoint regions.

## Introduction

**Fig. 1:**
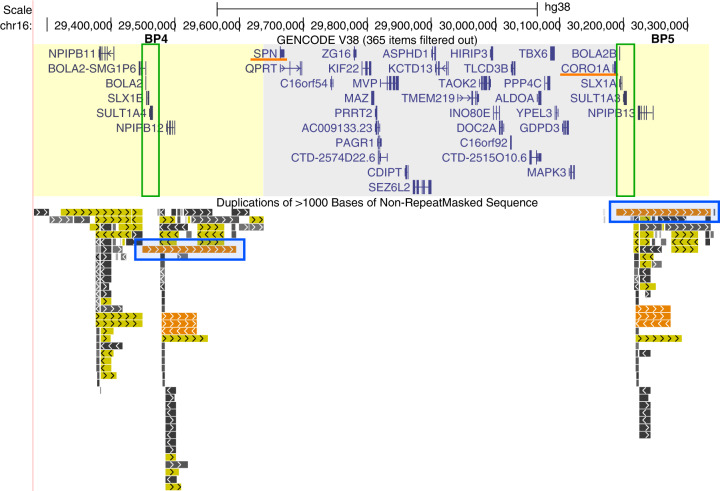
UCSC genome browser view of the 16p11.2 BP4-BP5 region. The GENCODE Genes (protein-coding genes, version 38) and Segmental Dups tracks are shown (genome assembly hg38). The single-copy region (~600 kbp in size, gray background) is present in *n* = 2 copies per diploid genome in euploid individuals, *n* = 1 copy in deletion carriers, and *n* = 3 copies in duplication carriers. Among the 30 protein-coding genes that map to this interval, *SPN* and *CORO1A* have known roles in leukocyte biology (underlined in orange). In the flanking segmental duplication clusters (yellow background), 10 protein-coding genes map and include paralogous copies of *NPIPB*, *BOLA2-SMG1P*, *BOLA2*, *SLX1*, and *SULT1A*. A high-identity duplication pair (highlighted by blue rectangles) generates the 16p11.2 BP4-BP5 CNV through non-allelic homologous recombination. The copy-number variant *BOLA2* duplicon (range: 3–8 copies per diploid genome) is marked by a green rectangle and contains *BOLA2/2B*, *SLX1A/B*, and *SULT1A3/4* genes. The scale bar corresponds to 500 kb.

Recurrent proximal 600 kbp long copy number variants (CNVs) at the 16p11.2 chromosomal region with breakpoints BP4 and BP5 (OMIM #611913 for the deletion and OMIM #614671 for the reciprocal duplication) are among the most frequent genetic causes of neurodevelopmental and psychiatric disorders^1–6^. These CNVs are also associated with mirror phenotypes on head circumference, body mass index, brain size, and pubertal onset^7–12^. They arise through non-allelic homologous recombination (NAHR) between paralogous copies of *Homo sapiens sapiens*-specific, directly-oriented, and copy-number polymorphic segmental duplications (or low-copy repeats, LCRs) that are located at both breakpoints^13^. The interval contains 40 protein-coding genes, i.e. 30 within the single-copy region from *SPN* to *CORO1A* and 10 (*BOLA2/2B*, *SLX1A/1B*, *SULT1A3/A4*, *NPIPB11/B12/B13*, and *BOLA2-SMG1P6*) mapping to the flanking segmental duplications^14^ (GENCODE v38, Fig. 1). Among segmental duplications at BP4 and BP5, a segment that contains the *BOLA2*, *SLX1*, and *SULT1A* genes (which we will refer to as the *BOLA2* duplicon) is present in six diploid copies in most humans (diploid copy number range: 3–8)^13^, while it shows an altered copy-number distribution in 16p11.2 BP4-BP5 deletion carriers as deletion alleles lose copies of this duplicon along with the intervening unique region^15^. We previously showed that reduced *BOLA2* copy number associates with anemia prevalence in 16p11.2 deletion carriers, suggesting that the disease-associated chromosomal instability accompanying these segmental duplications might be countered by the possibly advantageous increase of *BOLA2* copy number in improving systemic iron homeostasis^15^.

Besides the most frequently studied brain-related dysfunctions, additional morbidities, like iron-deficiency anemia^15,16^, genitourinary defects^12,17^, and abnormalities of the skeletal system^18,19^, have been found associated with gene dosage at the 16p11.2 locus. Such identification of new phenotypes has a medical relevance in the definition of the full clinical spectrum of 16p11.2 CNVs, as well as provides biological insights into the discovery of gene function in different tissues and organs. Vice versa, known functions of genes within the rearranged interval suggest biological systems that might be affected by 16p11.2 gene dosage and pinpoint to additional phenotypes that need to be investigated in CNV carriers.

The 16p11.2 BP4-BP5 interval contains several genes that are involved in leukocyte biology and are important for the proper functioning of the immune system. *SPN* (sialophorin, also known as *CD43*) encodes a sialylated glycoprotein on the cell surface of leukocytes that is involved in the regulation of multiple T cell functions, including activation, proliferation, differentiation, and trafficking^20^. *CORO1A* (coronin-1a) is abundantly expressed in leukocytes and is important in T lymphocyte trafficking^21^ and survival^22^. It has a crucial role in the immune response, as homozygous or compound heterozygous mutations of this gene are associated with immunodeficiency-8 (OMIM #615401), which is characterized by recurrent infections and T cell lymphopenia^23–25^. Among the families described to date, one presented with a *CORO1A* mutation on the paternal allele and a 16p11.2 deletion on the maternal allele^26^. *PAGR1* (PAXIP1 Associated Glutamate Rich Protein 1) encodes a protein that forms a complex with PAXIP1, a protein associated with thymocyte development^27^ and immunoglobulin class switch recombination^28^. *SLX1* encodes a protein that participates in a multiprotein complex together with SLX4. This complex plays a key role in the control of genome stability, DNA repair, homologous recombination, and telomere maintenance^29,30^. Although *SLX1* has never been associated with immunodeficiency, biallelic *SLX4* mutations were uncovered in patients with Fanconi anemia of complementation group P (OMIM #613951), a rare genetic disease characterized by bone marrow failure with inability to produce blood cells and high cancer susceptibility^31^. *SLX1* maps to the human-specific duplicon at the 16p11.2 locus and its copy number changes together with the one of *BOLA2*^13^. The BP4-BP5 region physically interacts with the neighboring BP2-BP3 interval^6^ that embeds the *LAT* gene with well-known functions in the immune system and a major contributor of the BP2-BP3 CNV-associated neuroanatomical phenotype^32^. Furthermore, genome-wide association studies (GWAS) of blood traits identified single-nucleotide variants in the 16p11.2 interval, suggesting a role of some 16p11.2 genes in eosinophil count phenotype^33–35^.

In this study, we investigated differential white blood cell (WBC) counts in 16p11.2 BP4-BP5 CNV carriers. The study group included both clinically ascertained CNV carriers and individuals from a population cohort. We uncovered an association between the 16p11.2 BP4-BP5 deletion and low lymphocyte count and a possible higher susceptibility to moderate neutropenia.

## Results

### 16p11.2 deletion carriers with lymphopenia and/or neutropenia in a 16p11.2 clinical cohort

We assessed clinical information and differential WBC counts of 32 unrelated 16p11.2 deletion carriers recruited by the 16p11.2 Consortium members (Supplementary Table 1). We classified two individuals with neutropenia and lymphopenia and investigated clinical and genetic information of the probands and other family members.

#### Family 1

**Fig. 2:**
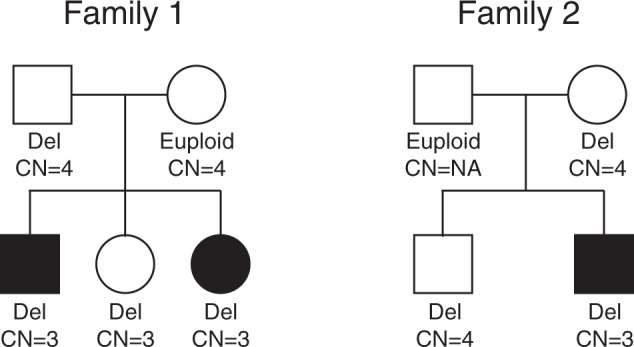
Pedigrees of two families with 16p11.2 deletion and neutropenia and low lymphocyte counts. The status for the 16p11.2 BP4-BP5 region (“Del” for deletion or “Euploid”) and the copy number (CN) of the *BOLA2* duplicon are shown. Filled symbols show members with low neutrophil and lymphocyte counts.

We identified a 14-year-old male proband (*Proband 1*) with a 16p11.2 deletion inherited from the father (Fig. 2). Blood test at the age of 5 years showed low hemoglobin and hematocrit levels, microcytosis, moderate neutropenia, and mild lymphopenia (Table 1). He underwent bone marrow examination that showed normal cytology and rich smears on which the different myeloid lines were correctly represented without noticeable morphological anomalies, nor blockage of maturation. No excess blasts or suspicious lymphoid cells were observed. Blood test at the age of 14 years showed a neutrophil count within the normal range and low lymphocyte count. His father (16p11.2 deletion carrier) and mother (16p11.2 euploid) showed neutrophil and lymphocyte counts within the normal ranges. His two sisters, 13 and 11 years old and both carriers of 16p11.2 deletion, showed, respectively, normal and low counts of neutrophils and lymphocytes (Table 1). The array-CGH did not identify any other CNV in this family besides the 16p11.2 deletion. The diploid copy number of the *BOLA2* duplicon was four in both parents and three in all kids. Deletion carriers of this family were receiving no drug therapy.

**Table 1:** Genetic and clinical information of two families with 16p11.2 deletion and neutropenia/lymphopenia.

Family	Individual	Gender	16p11.2 CNV status	*BOLA2* CN	Exome	Age blood test (years)	WBC (10^9^ cells/L blood)	NTR (10^9^ cells/L blood)	LYM (10^9^ cells/L blood)
1	Proband	M	Deletion carrier	3	1	5	**2.9**	**0.67**	**1.48**
						5	**2**	**0.22**	**1.14**
						5	**2.8**	**0.59**	1.54
						14	**4.3**	2.36	**1.41**
1	Mother	F	Euploid	4	1	44	5.7	3.08	2.13
1	Father	M	Deletion carrier	4	1	46	4.8	2.77	1.50
1	Sibling 1	F	Deletion carrier	3	0	13	**4.3**	2.24	1.67
1	Sibling 2	F	Deletion carrier	3	1	11	**3.3**	**1.54**	**1.30**
2	Proband	M	Deletion carrier	3	1	7	**3.2**	**0.83**	1.57
						8	**4.25**	**1.34**	1.79
						12	**2.86**	**0.58**	**1.19**
2	Mother	F	Deletion carrier	4	1	–	NA	NA	NA
2	Father	M	Euploid	NA	1	–	NA	NA	NA
2	Sibling	M	Deletion carrier	4	1	–	NA	NA	NA

*WBC* white blood cell counts, *NTR* neutrophil counts, *LYM* lymphocyte counts.

Values in bold are below the reference range (we considered for each individual the reference range reported by the laboratory where the analysis was performed. Generally, WBC counts are 4.5–11 × 10^9^ cells/L; neutrophil counts are 1.5–7 × 10^9^ cells/L; lymphocyte counts are 1.5–4 × 10^9^ cells/L).

To identify the possible existence of a variant explaining the low leukocyte count phenotype, we sequenced the exome of four family members (the proband, both his parents, and the younger affected sister). We assessed nonsynonymous exonic and splicing variants with a frequency lower or equal to 1% in gnomAD (v2.1.1) Exomes, gnomAD Genomes, and 1000 Genomes. We first focused our analysis on known genes associated with cytopenia. Comprehensively, we included 162 genes associated with the human phenotype ontology code HP_0001875 (“neutropenia”), 505 genes listed in the Genomics England panel “Primary immunodeficiency” (v. 2.421), 219 genes listed in the Genomics England panel “Cytopenias and congenital anemias” (v. 1.84), and 125 genes listed in the Genomics England panel “Cytopenia-NOT Fanconi anemia” (v.1.37), for an overlapping total of 730 genes (Supplementary Table 2). No filtered variants in these genes segregated in an autosomal recessive pattern (i.e. with heterozygous carrier parents and both siblings being either homozygous for the same variant or carrying compound heterozygous variants). We next considered a digenic model where the proband and his affected sister inherited the 16p11.2 deletion from the father and a second variant from the mother. We identified variants in *SLC34A1*, a sodium-phosphate transporter (rs145798898) and *EPHX1* (epoxide hydrolase, rs761149789) that were classified as benign by prediction tools (Table 2).

**Table 2:** Missense variants identified in Probands 1 and 2.

Proband	Gene	Inheritance pattern	Chr	Coordinate	Ref	Alt	Gen	AA change/splicing	dbSNP 150	PolyPhen-2 HDIV	PolyPhen-2 HVAR	CADD phred	SIFT	ClinVar	gnomAD Frequency	gnomAD N. homozygotes/hemizygotes	gnomAD Missense Z score
1	*SLC34A1*^a^	Digenic model	5	176813246	G	A	het	p.R95H	rs145798898	B	B	6.5	T	Benign	2.45E-03	4	−0.48
1	*EPHX1*^a^	Digenic model	1	226030139	C	T	het	p.T335M	rs761149789	B	B	11	T	–	4.60E-05	0	−0.06
1	*KIF11*	AR	10	94353214	G	C	het	splicing	rs200188195	–	–	–	–	Likely benign	6.64E-04	0	3.27
1	*KIF11*	AR	10	94413435	A	G	het	p.H1018R	rs147164679	B	B	14	T	–	5.69E-05	0	–
1	*PKP3*	Digenic model	11	394410	C	T	het	p.R40W	rs1052027375	P	B	25	D	–	1.37E-05	0	−0.73
1	*LIMS2*	Digenic model	2	128397844	C	T	het	splicing	–	–	–	28	–	–	0	0	0.37
1	*USP3*	Digenic model	15	63866277	T	G	het	p.F314C	–	D	D	30	D	–	0	0	2.11
2	*POLR3C*^a^	AR	1	145608556	C	T	hom	p.R84Q	rs115748902	D	B	–	T	Likely benign	4.84E-03	7	1.02
2	*ATP6AP1*^a^	X-linked recessive	X	153660787	G	A	hom	p.R180H	rs140841742	B	B	24	T	Benign	8.88E-04	46	1.53
2	*CFB*^a^	Digenic model	6	31919007	A	T	het	p.K648X	–	–	–	51	–	–	–	–	1.48
2	*MUC16*	AR	19	9059827	A	G	hom	p.S9207P	rs76869876	D	D	2	T_lc	–	8.55E-03	23	−8.06
2	*TLE2*	AR	19	3000655	C	T	het	p.S705N	rs143713547	D	D	25	T	–	5.87E-03	10	0.83
2	*TLE2*	AR	19	3011086	C	T	het	p.G316R	rs201317355	D	D	26	D	–	6.96E-03	9	–
2	*ZC3H3*	AR	8	144589985	G	A	het	p.S549L	rs149025999	P	B	26	D	–	3.45E-03	2	−0.01
2	*ZC3H3*	AR	8	144620334	C	G	het	p.K401N	rs145312531	P	B	25	D	–	4.11E-03	5	–
2	*C16orf92*	AR	16	30035111	G	A	hom	p.R65K	rs375771294	B	B	21	D	–	3.21E-05	0	0.16
2	*MAGEC1*	X-linked recessive	X	140992860	T	C	hom	p.M1T	rs113574601	P	B	1.6	D_lc	–	3.51E-03	262	−5.17
2	*CCNQ*	X-linked recessive	X	152860096	C	T	hom	p.R113H	rs150562029	B	B	16	T	Likely benign	2.19E-03	173	1.14
2	*CHD1L*	AD	1	146757100	G	A	het	p.A652T	–	B	B	21	D	–	0	0	−0.58

*Gen* genotype in the proband; *SIFT: T* tolerated, *D* deleterious, *T_lc* tolerated low confidence, *D_lc* deleterious low confidence; *PolyPhen-2: B* benign, *P* possibly damaging, *D* probably damaging.

Exonic nonsynonymous and splicing variants with frequency < 1% in gnomAD Exomes, gnomAD Genomes, and 1000 Genomes. Variant frequency, number of homozygotes and hemizygotes, and missense Z scores from gnomAD v2.1.1.

^a^Known cytopenia genes.

We then extended the analysis to the entire exome and identified compound heterozygous variants (rs200188195 and rs147164679) in *KIF11* (kinesin family member 11) in both siblings. The former is a rare splicing variant classified as likely benign in ClinVar; the latter is a rare missense variant with uncertain significance. We also specifically checked for rare variants in the 16p11.2 BP4-BP5 region but did not find any. Lastly, we considered a digenic model and assessed heterozygous variants inherited from the mother. We found an exonic nonsynonymous variant in *PKP3* (plakophilin 3, rs1052027375, CADD = 25.2, predicted as deleterious by SIFT) and a splicing variant in *LIMS2*, coding a focal adhesion protein (NM_001136037:exon9:c.868 + 1 G > A, CADD = 28.3). Both genes were associated with lymphocyte counts in genome-wide association studies^35,36^. We also identified a variant in *USP3*, a ubiquitin-specific peptidase.

#### Family 2

The second case was a male (*Proband 2*) with a 16p11.2 deletion inherited from the mother and no additional CNV shown by array-CGH (Fig. 2). Blood tests at the age of 7 and 12 years showed, respectively, mild and severe neutropenia with low lymphocyte counts (Table 1). The study of lymphocyte subpopulations showed lower values of T, B, and NK lymphocytes. The myelogram was normal and showed rich smears without excess blasts, dystrophy on the different lines, or abnormal cells. He had diminution of beta and gamma globulins. He was not receiving any medication. His euploid father had a moderate deficit in von Willebrand factor. No hematological problems were reported for his mother and brother, both 16p11.2 deletion carriers; however, detailed hematological values of the rest of the family were not available. The copy number of the *BOLA2* duplicon was three in the proband and four in his mother and brother.

We looked for potential causative variants for the leukopenia by exome sequencing the proband, his parents, and his unaffected brother. We applied the same filters as in the analysis of *Family 1*. We looked for recessive (homozygous or compound heterozygous), X-linked (hemizygous state in the proband and heterozygous or absent in the rest of the family), and dominant variants (present in the proband and absent in the rest of the family). We also considered a digenic model where the proband inherited the 16p11.2 deletion from the mother and a second variant from his father. Within the 730 cytopenia genes, whereas we found no de novo variant, we identified a homozygous variant in *POLR3C* (RNA polymerase III subunit C, p.R84Q) and a hemizygous variant in *ATP6AP1*, coding an accessory subunit of the vacuolar ATPase that mediates acidification of secretory vesicles, on the X chromosome (p.R180H). Both variants are predicted to be benign/tolerated. When we considered a digenic model, we identified a stop gain variant in *CFB* (complement factor B, LOEUF = 0.52) inherited from the father (p.K648X). Next, we extended the analysis to the whole exome and identified a homozygous variant in *MUC16* (mucin 16, cell surface associated, p.S9207P); compound heterozygous variants in *TLE2*, a transcriptional corepressor, and *ZC3H3*, coding a CCCH-type zinc-finger protein that regulates mRNA nuclear adenylation and export^37^; hemizygous variants in the X chromosome genes *MAGEC1* and *CCNQ* (cyclin Q); a variant in the 16p11.2 gene *C16orf92* inherited from the father (p.R65K); and a de novo variant in *CHD1L*, coding a DNA helicase involved in DNA repair. However, we did not identify any evidence in the literature for a role of these genes in hematological phenotypes. All filtered variants and associated predictions of deleteriousness are listed in Table 2.

Besides these two probands, we identified two individuals with low lymphocyte counts (<1.5 × 10^9^/L of blood): a male with three and a female with five copies of the *BOLA2* duplicon. Differently from *Probands 1* and *2*, the low lymphocyte count is based on a single blood measurement and they never received a diagnosis of neutropenia.

As both neutropenic probands had the lowest copy number of the *BOLA2* duplicon (i.e. three copies), we assessed a possible association between the neutropenia/lymphopenia and the duplicon copy number. Among the 32 unrelated 16p11.2 deletion carriers, 9 had 3 copies, 13 had 4, 10 had 5, and one had 8 (Supplementary Table 1). We did not find a significant enrichment of neutropenia (Fisher’s exact test, *P* = 0.07) or lymphopenia (Fisher’s exact test, *P* = 0.06) among deletion carriers with three copies of the duplicon *versus* those with more than three copies.

### Effects of 16p11.2 copy number variation on white blood cell counts and disease in a population cohort

**Fig. 3:**
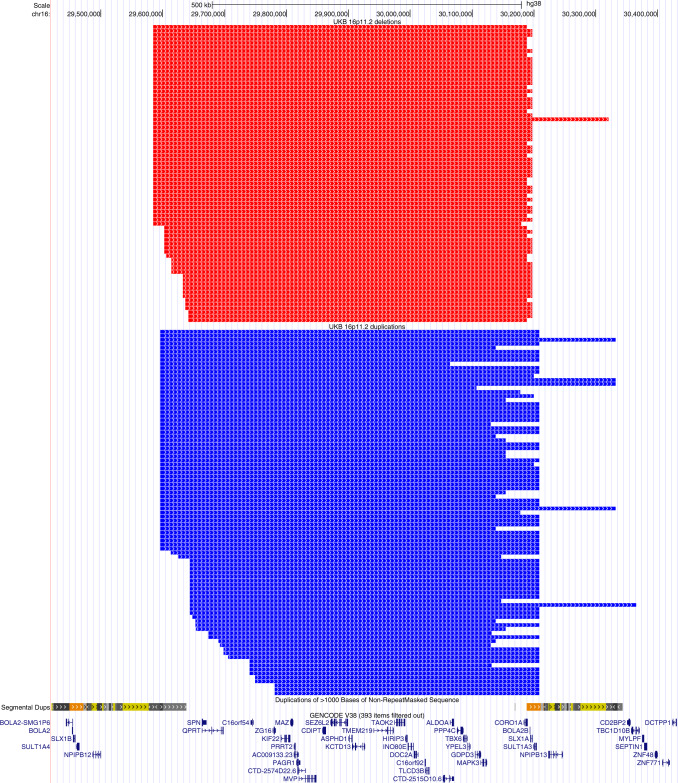
UCSC genome browser view of 16p11.2 CNVs identified in individuals from UKB. The Segmental Dups and GENCODE Genes (protein-coding genes, version 38) tracks are shown. All deletions (red blocks) encompass the whole single-copy region (from *SPN* to *CORO1A*) that is flanked by segmental duplication clusters where there is uncertainty about the exact position of the breakpoints. As expected, breakpoints of duplications (blue blocks) are more variable, reflecting the greater difficulty in detecting duplications than deletions using SNP-array data^61^. However, because of the NAHR mechanism that generates CNVs at this locus, we would expect that all deletion and duplication breakpoints fall within the flanking segmental duplication clusters (BP4 and BP5 regions).

As we identified no rare deleterious/damaging variant segregating with lymphopenia and/or neutropenia in either families, we assessed whether these phenotypes could be associated with 16p11.2 deletion and had an incomplete penetrance. We interrogated data from the UK biobank (UKB) adult population cohort where we identified 74 (29 females and 45 males) 16p11.2 deletion and 91 (48 females and 43 males) duplication carriers (Fig. 3). We used linear regressions to test the association of WBC differential counts (WBC count, neutrophil count, lymphocyte count, monocyte count, eosinophil count, and basophil count) with copy number of the 16p11.2 region (Methods, Table 3). Contrary to the neutropenia identified in the two families, deletion carriers had increased neutrophil counts (deletion versus controls: beta = 0.53, *P* adjusted = 8.31e-05) and no deletion carriers had a neutrophil count <1.5 × 10^9^/L of blood, the threshold for neutropenia. Deletion carriers also had significantly lower lymphocyte counts (deletion versus controls: beta = −0.35, *P* adjusted = 0.04), in line with the lymphopenia observed in the clinically ascertained probands, and higher basophil counts (deletion versus controls: beta = 0.32, *P* adjusted = 0.03) than controls. As medication use might impact WBC counts, we included this information as covariates. We found that all three associations remained (neutrophil count: beta = 0.38, *P* adjusted = 3.25e-3; lymphocyte count: beta = −0.37, *P* adjusted = 5.04e-3; basophil count: beta = 0.29, *P* adjusted = 2.31e-2), although the decreased association strength with neutrophil count might indicate partial confounding by drug usage.

**Table 3:** White blood cell differential counts in 16p11.2 CNV carriers from the UK biobank and comparison with controls.

Model	Count	Beta	SE	*t*	*P*	*P* adjusted
Del vs. controls	WBC	0.312	0.118	2.642	0.01	0.10
Del vs. controls	Neutrophil	0.531	0.118	4.496	6.93E-06	**8.31E-05**
Del vs. controls	Lymphocyte	−0.348	0.118	−2.959	3.09E-03	**0.04**
Del vs. controls	Monocyte	−0.003	0.114	−0.029	0.98	1.00
Del vs. controls	Eosinophil	−0.233	0.116	−2.001	0.05	0.54
Del vs. controls	Basophil	0.323	0.108	2.998	2.71E-03	**0.03**
Dup vs. controls	WBC	−0.029	0.107	−0.276	0.78	1.00
Dup vs. controls	Neutrophil	−0.052	0.107	−0.487	0.63	1.00
Dup vs. controls	Lymphocyte	0.054	0.106	0.508	0.61	1.00
Dup vs. controls	Monocyte	−0.175	0.103	−1.689	0.09	1.00
Dup vs. controls	Eosinophil	0.024	0.105	0.228	0.82	1.00
Dup vs. controls	Basophil	0.011	0.097	0.117	0.91	1.00

Adjusted *P* < 0.05 are in bold.

We then evaluated the presence of low WBC count disease among 16p11.2 CNV carriers. We identified two deletion carriers, one male and one female (2.7%), one duplication carrier, and 3221 control individuals (1%) who received a diagnosis of agranulocytosis/neutropenia (ICD-10 D70). Of note, the neutropenia in the female deletion carrier could be secondary as she had multiple myeloma (ICD-10 C900) and received chemotherapy (ICD-10 Z511). Overall, we were unable to pinpoint a link between 16p11.2 deletion and low WBC count disease in UKB (Fisher’s exact test, *P* = 0.1).

### Decreased expression of *CORO1A*, *KIF22*, and *BOLA2-SMG1P6* significantly associates with decreased lymphocyte counts

We hypothesized that the most plausible genetic mechanism for the observed phenotypes is a reduced expression of some genes within the 16p11.2 region as a consequence of the deletion. To pinpoint which genes could be causative of the associations observed using the UKB data (16p11.2 deletion with increased basophil and neutrophil counts and decreased lymphocyte counts) through gene expression, we conducted Mendelian randomization (MR) studies^38^. Among 82 genes within the 16p11.2 interval (Ensembl genes annotated in the interval hg38_chr16: 29303418-30337671 listed in Supplementary Table 3), 26 had at least one expression quantitative trait locus (eQTL) and could be assessed by MR. We tested 156 (26 × 6) gene-trait pairs and identified 22 significant associations (threshold for significance: 0.05/(26 × 6) = 3.2e-4) that passed the heterogeneity filter (Supplementary Table 4 and Methods section). While we found no significant association with basophil and neutrophil counts, we found that decreased expression of *CORO1A* (beta = 0.14, *P* = 2e-15), *KIF22* (beta = 0.16, *P* = 6e-6), and *BOLA2-SMG1P6* (beta = 0.03, *P* = 1e-13) significantly associated with decreased lymphocyte counts. Of note, these genes could be instrumented by only one or two genetic variants, which is insufficient to assess robustness of the effect estimates through further sensitivity analyses.

### Mouse models of 16p11.2 deletion and *Slx1b* deficiency and haploinsufficiency show no overt leukocyte count phenotypes

In our previous work^15^, we measured hematological parameters in male and female 16p11.2^Del/+^ (Del/+) and 16p11.2^Dup/+^ (Dup/+) mouse models^39^ at different ages. We repurposed these data to assess in a model organism the association between gene dosage at the 16p11.2 orthologous locus and leukocyte differential counts. The neutrophil count was not different between the Del/+ or Dup/+ mice compared to their wild-type littermates at any age point. Similarly, the lymphocyte count was largely not different between the groups.

As *SLX1*, which maps to the human-specific and copy-number variant duplicon, might be associated with the low WBC count in 16p11.2 deletion carriers, we analyzed the hematology of *Slx1b* haploinsufficient and deficient mice. We measured 44 hematological parameters in 41 mice of both sexes and all three genotypes (from 4 to 9 mice per condition). The age of mice at the time of assessment was within the range of 9–11 weeks. We compared HET vs WT and HOM vs WT mice, by gender, and found no significant difference in any parameter after multiple test correction (Supplementary Table 5).

## Discussion

In this study, we evaluated leukocyte differential counts and disease, particularly lymphopenia and neutropenia, in 16p11.2 CNV carriers as some genes mapped to the rearranged region have known roles in leukocyte biology. Lymphopenia is defined as an absolute lymphocyte count <1.5 × 10^9^/L in adults and <2.0 × 10^9^/L in children^40^. Neutropenia is defined by a decrease in the number of neutrophilic granulocytes circulating in the blood and may be mild (count between 1.0 and 1.5 × 10^9^/L), moderate (count between 0.5 and 1.0 × 10^9^/L), or severe (count <0.5 × 10^9^/L)^40^. Among white British control individuals from UKB, 0.35% (1163/335789) have a neutrophil count <1.5 × 10^9^ cells/L of blood and 1% (3221/335789) received a diagnosis of neutropenia, similar to the prevalence of 0.5% previously reported for adult white individuals^41^. Congenital neutropenia, i.e. chronic neutropenia caused by a constitutional genetic defect, is a rare and genetically heterogeneous disease with a prevalence of ~1/100,000 people^42^. Whereas mutations in 24 different genes were shown to be associated following a dominant, recessive, or X-linked inheritance pattern, more genes need to be identified as the genetic origin remains unknown in 30% of congenital neutropenia cases^43^.

Among 32 unrelated 16p11.2 deletion carriers for which we had blood cell count information, we identified two cases with lymphopenia and neutropenia. Both probands were also included in our previous study and had the lowest copy number of the *BOLA2* duplicon that we previously associated with anemia^15^, and while Proband 1 was anemic, Proband 2 was not. These individuals had a normal bone marrow cytology and no record of recurrent infections. Exome investigation of both families revealed no deleterious variants in known cytopenia genes that could explain the low leukocyte phenotype. In our analysis of Proband 2 that inherited the 16p11.2 deletion from his mother, when we considered a digenic model, we identified a paternal stop gain variant in *CFB* (complement factor B). Biallelic *CFB* mutations cause complement factor B deficiency (#615561), an autosomal recessive disease characterized by recurrent infections of encapsulated organisms with normal lymphocyte subsets^44^. We therefore think that the loss of function of the paternal *CFB* gene is probably not associated with the low leukocyte counts. In the exome-wide analysis of Family 1, we identified maternally inherited variants in *PKP3*, encoding a member of the armadillo protein family that participates in immune response regulation^45^, and *USP3* (ubiquitin Specific Peptidase 3), a gene expressed in the bone marrow and white blood cells whose deficiency affects hematopoiesis with progressive loss of B and T cells in mice^46^. It is possible that the predicted deleterious variant in *USP3* could act as second hit to determine the lower leukocyte counts. In our exome-wide analysis of Proband 2, we identified a paternally inherited rare missense variant in the 16p11.2 gene *C16orf92* that encodes an uncharacterized protein and likely benign rare missense variants in other genes that have no known roles in leukocyte biology.

Among 74 deletion carriers from the UKB adult population cohort, we identified one individual with neutropenia and a second case where the neutropenia could be secondary to chemotherapy. The identification of 4/106 16p11.2 deletion carriers with neutropenia (two clinically recruited and two from UKB) might suggest incomplete penetrance for neutropenia (around 3–4% in deletion carriers versus 0.5–1% in non-carriers). However, UKB 16p11.2 deletion carriers also showed higher neutrophil counts, thus arguing against a role of 16p11.2 deletion for neutropenia. Possible explanations for this discrepancy are: (1) both associations are true and there is an age effect on neutrophil count in 16p11.2 deletion, as in both families affected individuals are young, while the UKB is an adult population cohort (40–69 years); (2) clinically-recruited neutropenic 16p11.2 deletion carriers carry another causative genetic variant that cannot be identified through exome sequencing; and (3) other confounding factors—medication use excluded—are at play and drive the associations.

MR analyses, that were limited by the availability of gene-associated eQTLs, pinpointed to decreased expression of *CORO1A*, *KIF22*, and *BOLA2-SMG1P6* as potentially causative of the lower lymphocyte counts. *CORO1A* involvement is consistent with previous reports of lymphopenia in carriers of homozygous or compound heterozygous mutations of this gene^23–25^. *BOLA2-SMG1P6* association had a lower strength and suggests a possible additional role of the flanking segmental duplications where this gene is located. However, when applied to duplicated genes, MR analyses have some limitations as few SNPs are annotated in segmental duplications and expression of genes embedded to these loci is poorly quantified because of short-read mapping issues to multiple highly similar sequences and gene annotation errors. MR analyses identified no causative gene for the different neutrophil and basophil counts.

We also hypothesized that, due to its role in the SLX1-SLX4 complex, a low copy number of *SLX1* could contribute to the low leukocyte counts, although with incomplete penetrance. We thus assessed mouse models of *Slx1* haploinsufficiency and deficiency, but these did not show lower counts of any white blood cell type. This lack of phenotype in mice may indicate that *SLX1* is not a responsible gene. However, it is still possible that *SLX1* has a role but shows a human-specific phenotype and/or the copy number and structural organization of the human-specific duplicon is relevant, for example by affecting the expression of the adjacent *CORO1A*. Regarding the human-specificity, WBC counts are known to be variable across species. For example, humans have lower WBC counts than great apes, derived from lower counts of both neutrophils and lymphocytes^47^, and while humans and great apes have higher number of neutrophils than lymphocytes per mL of blood, in mouse this ratio is opposite. In favor of a potential role of *SLX1*, an intergenic variant (rs7947929) upstream of *MUS81*, encoding another member of the SLX1-SLX4 complex, was shown to be associated with neutrophil count^36^ and to be an eQTL of *MUS81* in several tissues, including whole blood (GTEx Portal)^48^.

In conclusion, our data suggest that 16p11.2 deletion, and possibly also the lowest dosage of the human-specific *BOLA2* duplicon, are associated with low lymphocyte counts. We found a trend between lower copy number of the *BOLA2* duplicon and higher susceptibility to moderate neutropenia among 16p11.2 deletion carriers. However, the description of further deletion carriers with lymphopenia and/or neutropenia are warranted to clarify these associations.

## Methods

### Phenotyping and *BOLA2* copy number genotyping in the 16p11.2 clinical cohort

The Committee of Human Research Ethics of the Canton of Vaud, Switzerland, approved the project. Participants of the 16p11.2 clinical cohort were recruited based on direct or cascade diagnosis of 16p11.2 CNV carrier status in probands and their families. We obtained written informed consent from clinical cohort participants to perform this study. Hematological data of 16p11.2 deletion probands and other family members were collected by the respective physicians. The copy number of the *BOLA2* segment in 16p11.2 BP4-BP5 deletion carriers was estimated with a molecular inversion probe (MIP) assay as described in Nuttle et al.^49^ and Nuttle et al.^13^. Briefly, we designed 47 MIPs, i.e. single-stranded DNA molecules containing two regions complementary to sequences in the *BOLA2* segment that flank informative paralogous sequence variants. We then sequenced the amplification products on a MiSeq platform and estimated paralog-specific copy number genotypes of proximal (BP5) and distal (BP4) segments as well as aggregate copy number genotypes based on sequencing read depth, though the former can be confounded if there is gene conversion affecting the informative variants. We calculated a LOD score to evaluate the genotype confidence by comparing likelihoods of the MIP data between the called genotype and the next most confident genotype.

### Exome sequencing and analysis

Genomic DNA was extracted from peripheral blood by the medical genetics service referring each 16p11.2 deletion carrier using locally established standard protocols. Concentration and quality of the DNA samples were quantified using Qubit. Sample quality and integrity was further evaluated using NanoDrop spectrophotometer and agarose gel. After passing quality requirements, exome sequencing was performed at the Lausanne Genomic Technologies Facility. Genomic libraries were prepared using the SureSelect Exome V5 kit from Roche or the IDT’s (Integrated DNA Technologies) xGen Exome Research Panel and sequenced as 100 bp paired-end reads on Illumina HiSeq2500 platform. Mean bait coverage ranged from 81 to 275 across samples. Exome data were analyzed as previously described^50^. Purity-filtered reads were adapters and quality trimmed with FastqMcf v. 1.1.2^51^ and aligned to the human_g1k_v37_decoy genome using BWA-MEM v. 0.7.10^52^. PCR duplicates were marked using Picard tools v. 1.130 (http://broadinstitute.github.io/picard/). BAM files were further processed with GATK v. 3.3 for realignment around indels and base quality score recalibration^53^. Variant calling and quality filtering were performed on all samples using GATK HaplotypeCaller in gVCF mode and GATK^54^. Exome variant filtering and analysis was performed using the VarAFT tool^55^. The potential impact of nonsynonymous coding variants was assessed using the Polymorphism Phenotyping v2 (PolyPhen-2)^56^, Sorting Intolerant From Tolerant (SIFT)^57^, and Combined Annotation Dependent Depletion (CADD)^58^ scores.

### CNV calling and statistical analyses in UK Biobank population cohort

The UK Biobank (UKB)^59^ is a volunteer-based general population biobank of the United Kingdom. Half a million participants aged 40–69 years at the time of recruitment (2006–2010) were enrolled through National Health Service registers. Participants consented to provide personal and health-related information, biological samples, and to have their DNA tested. The UKB governing Research Ethics Committee has approved a generic Research Tissue Bank approval to the UKB, which covers the research using this resource. CNV calling was performed as described in Auwerx, et al.^60^. We called CNVs using PennCNV^61^ and attributed to CNV calls a quality score^62^. We considered “16p11.2 BP4-BP5 CNV carriers” those that had a deletion or duplication starting in the interval 29.4–29.8 Mbp and ending 30.05–30.4 Mbp (hg19) and retained high confidence CNV calls (|QS| ≥ 0.5). We restricted the analysis to 335′954 unrelated participants who declared themselves as white British.

We searched for evidence of association between gene dosage at 16p11.2 BP4-BP5 (deletion carriers, control individuals, and duplication carriers) and hematological traits relative to white blood cells (WBC count, neutrophil count, lymphocyte count, monocyte count, eosinophil count, and basophil count), using linear models in the statistical package R^63^. We considered two models: (i) deletion versus control (del = 1, ctrl = 0); and (ii) duplication versus control (dup = 1, ctrl = 0). Trait measures were normalized by rank-base inverse normal transformation. We included as covariates age, age^2^, sex, and the first 40 principal components from the genetic analysis. We performed the analysis including all individuals together and dividing them by sex. We tested for sex-specific effects for those traits with *P*_*all*_ ≤ 0.05 using Eq. (1).

1 $$t = \frac{{\beta _{{\mathrm{male}}} - \beta _{{\mathrm{female}}}}}{{\sqrt {SE_{{\mathrm{male}}}^2 + SE_{{\mathrm{female}}}^2} }}$$

As we found no evidence for sex-specific effects, we continued the analyses considering only all individuals together. We considered as significant those associations with Bonferroni multiple test correction significance *P* ≤ 0.05, i.e. associations with a nominal *P* ≤ 0.05/12 = 0.0042, as we adjusted for the multiple testing of six independent traits and two models.

We next checked whether the three significant associations were confounded by medication use. We used treatment/medication use (data-field 20003) collected for UKB participants, added the ATC (Anatomical Therapeutic Chemical) 3rd level code to drug names^64^, and selected 70 ATC categories used by at least one 16p11.2 CNV (deletion or duplication) carrier. We added these data as covariates in the regression analysis. As we performed three tests, we considered *P* ≤ 0.05/3 as significant.

We used both primary and secondary hospital discharge diagnoses (data-field 41270) collected for UKB participants. We assessed the presence of ICD-10 (International Statistical Classification of Diseases and Related Health Problems, 10th revision) code D70 (neutropenia).

### Mendelian randomization

We performed Mendelian randomization (MR) analyses^38^ to assess whether expression of genes in the 16p11.2 region (Supplementary Table 3) was causally associated with six investigated WBC traits. MR analyses allowed to estimate the causal effect of an exposure (i.e. gene expression) on an outcome (i.e. WBC trait) based on independent (r^2^ < 0.01) instrumental variables (IVs) and their genetic effect sizes on these two quantities. The effect sizes on gene expression came from eQTL data derived from whole blood as provided by the eQTLGen consortium (cis-eQTLs, FDR < 0.05, 2-cohort filter)^65^. Effect sizes on WBC traits came from GWAS summary statistics conducted on the UK Biobank (basophil (#30160), eosinophil (#30150), lymphocyte (#30120), monocyte (#30130), neutrophil (#30140), and WBC (#30000) counts; http://www.nealelab.is/uk-biobank/). Prior analyses, eQTL and GWAS data were harmonized, palindromic SNPs were removed, as were SNPs with an allele frequency difference > 0.05 between datasets. Significance of the MR estimates was defined by a *P* ≤ 0.05/(26 × 6) = 3.2e-4, to account for the testing of 26 genes with at least one IV and six traits. For genes with ≥3 IVs, we assessed the robustness of the MR effect estimates by performing heterogeneity tests based on Cochran’s Q-statistic^66^. Homogeneity of the IVs was assured through a heterogeneity *P* ≥ 0.01.

### Mouse models and hematological measurements

We measured 44 hematological parameters in 16p11.2^Del/+^ and 16p11.2^Dup/+^ male and female mouse models, as well as their respective wild-type littermates, at 7, 15, 29, and 50 weeks of age (5–12 mice per group)^15^.

The Slx1b^em1(IMPC)Ics^ allele was produced in the frame of the International Mouse Phenotyping Consortium^67^ on the C57BL/6N background using the CRISPR/Cas9 system. The sperm of heterozygous mice was acquired from the Institut Clinique de la Souris, Illkirch, France and the line was recovered at the University of Lausanne, Switzerland through in vitro fertilization of oocytes from wild-type C57BL/6N females. The animals were maintained on a standard chow diet (Kliba 3436, 250 ppm iron), provided water and food ad libitum and housed in 12-h-long cycles of light and darkness. Whole blood was collected from the tail vein in EDTA tubes (Sarstedt) for hematological measurements that were executed by the Centre de PhénoGénomique, EPFL, Lausanne as described in Giannuzzi et al.^15^. All procedures were performed in accordance with protocols approved by the cantonal veterinary authority. We generated a cohort by setting ko (knock-out)/ + x ko/ + breedings. Mice of both sexes and with genotypes +/+ (WT), ko/+ (HET), and ko/ko (HOM) were born following a Mendelian distribution. WT, HET, and HOM mice showed no weight difference between 8 and 11 weeks of age (data not shown).

We used t-tests (two-sided) to assess differences in hematological traits between HET and HOM *Slx1b* knockout versus wild-type mice. *P* values were adjusted using the Benjamini & Hochberg correction, considering 176 tests (44 parameters, two genders, two models). Statistical analyses were performed using the R software environment^63^.

### Reporting summary

Further information on research design is available in the Nature Research Reporting Summary linked to this article.

## Supplementary information

Reporting Summary

Data Set 1

Data Set 2

Data Set 3

Data Set 4

Data Set 5

## Data Availability

The identified variants have been submitted to ClinVar under accession numbers SCV002505380 and SCV002507121–SCV002507139. Exome sequencing data are available from the corresponding author upon reasonable request. The data are not publicly available due to privacy issues and access will require the signing of a Data Usage Agreement.

## Code availability

Exome data analysis was performed using VarAFT version 2.17-1. Code for the MIP assay is available on request. Code for the CNV association with WBC phenotypes is available at https://github.com/cauwerx/16p11.2_wbc. Code and detailed documentation to conduct Mendelian randomization analyses of gene expression on traits are hosted at https://github.com/masadler/smrivw.
