# Author Correction: Possible association of 16p11.2 copy number variation with altered lymphocyte and neutrophil counts

**DOI:** 10.1038/s41525-023-00354-z

**Published:** 2023-05-24

**Authors:** Giuliana Giannuzzi, Nicolas Chatron, Katrin Mannik, Chiara Auwerx, Sylvain Pradervand, Gilles Willemin, Kendra Hoekzema, Xander Nuttle, Jacqueline Chrast, Marie C. Sadler, Eleonora Porcu, 16p11.2 Consortium, Katrin Männik, Damien Sanlaville, Caroline Schluth-Bolard, Cédric Le Caignec, Mathilde Nizon, Sandra Martin, Sébastien Jacquemont, Armand Bottani, Marion Gérard, Sacha Weber, Aurélia Jacquette, Catherine Vincent-Delorme, Aurora Currò, Francesca Mari, Alessandra Renieri, Alfredo Brusco, Giovanni Battista Ferrero, Yann Herault, Bertrand Isidor, Brigitte Gilbert-Dussardier, Evan E. Eichler, Zoltan Kutalik, Alexandre Reymond

**Affiliations:** 1 Center for Integrative Genomics, University of Lausanne, Lausanne, Switzerland; 2 Department of Biosciences, University of Milan, Milan, Italy; 3 Service de génétique, Hospices Civils de Lyon, Lyon, France; 4 University of Lyon, Université Claude Bernard Lyon 1, CNRS UMR-5310, INSERM U-1217, Institut NeuroMyoGène, F-69008 Lyon, France; 5 Department of Computational Biology, University of Lausanne, Lausanne, Switzerland; 6 Center for Primary Care and Public Health, University of Lausanne, Lausanne, Switzerland; 7 Swiss Institute of Bioinformatics, Lausanne, Switzerland; 8 Department of Genome Sciences, University of Washington, Seattle, WA USA; 9 Center for Genomic Medicine, Massachusetts General Hospital, Boston, MA USA; 10 Department of Neurology, Harvard Medical School, Boston, MA USA; 11 Program in Medical and Population Genetics and Stanley Center for Psychiatric Research, Broad Institute, Cambridge, MA USA; 12 University of Strasbourg, CNRS, INSERM, PHENOMIN-ICS, Institute of Genetics and Molecular and Cellular Biology, Illkirch, France; 13 Service de Génétique Médicale, CHU de Nantes, Nantes, France; 14 Service de Génétique, CHU de Poitiers, Poitiers, France; 15 Howard Hughes Medical Institute, University of Washington, Seattle, WA USA; 16 Centre Hospitalier Universitaire Vaudois, University of Lausanne, Lausanne, Switzerland; 17 University of Montreal, Montreal, Canada; 18 Department of Genetic Medicine and Development, University of Geneva Medical School, Geneva, Switzerland; 19 Service de Génétique Medicale, CHU de Caen, Caen, France; 20 Service de Génétique Clinique, CHU Paris-GH La Pitié Salpêtrière, Paris, France; 21 Service de Génétique Clinique, CHU de Lille, Lille, France; 22 Medical Genetics, University of Siena, Siena, Italy; 23 Department of Medical Sciences, University of Turin, Turin, Italy; 24 Department of Clinical and Biological Sciences, University of Turin, Turin, Italy; 25 Present Address: Health 2030 Genome Center, Foundation Campus Biotech, Geneva, Switzerland

**Keywords:** Haematological diseases, Genetic association study, Medical genetics

Correction to: *npj Genomic Medicine* 10.1038/s41525-022-00308-x, published online 17 June 2022

In the Acknowledgements section, the below content was missing:

“This research has been supported (not financially) by European Reference Network for rare malformation syndromes and rare intellectual and neurodevelopmental disorders, ERN-ITHACA [EU Framework Partnership Agreement ID: 3HP-HP-FPA ERN-01-2016/739516]. This ERN is partly co-funded by the European Union within the framework of the Third Health Programme “ERN- 2016 - Framework Partnership Agreement 2017-2021”.

The original article has been corrected.

